# Is there a “mind” behind the music? Attributing music to AI can suppress narrative meaning-making

**DOI:** 10.1186/s41235-026-00715-z

**Published:** 2026-03-02

**Authors:** Sarah H. Wu, Kevin J. Holmes

**Affiliations:** 1 Department of Psychology, Reed College, 3203 SE Woodstock Blvd, Portland, OR 97202-8199, USA; 2 Department of Communication, Stanford University, 450 Jane Stanford Way Bldg. 120, Stanford, CA 94305-2050 USA

**Keywords:** Narrative listening, AI, music, meaning-making

## Abstract

The rise of AI-generated music has implications for how people derive meaning from the listening experience, including the propensity to imagine a story as music unfolds. Previous research suggests that such *narrative listening* requires some form of common ground between composer and listener. Therefore, people may be less likely to engage in narrative listening when they believe music is the product of an AI system rather than a human mind. We tested this possibility across two preregistered studies in which US participants (*N* = 399) listened to several pieces of instrumental music and reported their experience of narrative listening—whether they imagined a story and how engaging it was. When presented with unlabeled, human-composed music, participants reported imagining fewer and less engaging narratives in response to pieces they regarded as more likely computer generated than human composed (Study 1). When we experimentally manipulated the purported composer by labeling human- and AI-composed music clips as either “Human” or “AI” composed, the “AI”-labeled pieces elicited fewer and less engaging narratives than their “Human”-labeled counterparts, regardless of the actual composer (Study 2). Together, these findings suggest that ascribing music to AI is associated with—and can engender—an impoverished listening experience, devoid of the mental narratives that unfold as the composer’s musical choices guide the listener’s imagination. Our findings contribute to an emerging literature on perceptions of artificial creators, with practical implications for listeners, musicians, and policymakers.

**Supplementary Information:**

The online version contains supplementary material available at 10.1186/s41235-026-00715-z.

## Significance statement

To the dismay of many artists and audiences, the use of generative artificial intelligence (AI) has become more and more common in creative fields—historically regarded as uniquely human. Despite the impressive and convincingly human outputs of creative AI systems, audiences may fail to connect with artistic work if they simply *believe* it is the product of a machine rather than a human mind. Our studies provide evidence for this diminished sense of connection in the context of listening to music. We found that listeners of instrumental music were less likely to imagine a story while listening—and the stories they imagined were less engaging—when they believed the music was AI generated, even when it was actually human composed. This suggests that the depth of meaning people derive from music depends not only on how the music sounds or the common understandings it evokes, but also on beliefs about its creator. Our findings highlight a key limitation of AI in the arts: Even though AI systems can produce works that look or sound impressive, audiences may engage with them in a rather shallow way, missing the human touch that makes art feel meaningful. By the same token, falsely framing AI-generated art as a human creation may elicit a greater sense of meaning—but at a cost to human creators, depriving them of credit and compensation for the work from which AI products are derived.

## Introduction

In 2025, the band The Velvet Sundown rose to prominence with over a million monthly listeners on Spotify. Against predictable chord progressions, a male voice sings vaguely of “dust on the wind.” Neither engaging nor unpleasant, the song makes for an adequate road-trip soundtrack. Click on the hazy album cover photograph and you’ll learn that the band is in fact a “synthetic music project guided by human creative direction,” with its songs, album art, and social media presence all produced by artificial intelligence (AI). As one listener put it, each generic lyric “[r]eally makes you think, until you realize that, no, it doesn’t at all” (Bogost, 2025).

The rise of AI-generated music has implications for how people derive meaning from the listening experience. One route to meaning-making is to engage with music as if it were a story. Even in the absence of lyrics, listeners routinely interpret music as not just a series of delimited pitches, but a narrative complete with characters, events, and emotions—a phenomenon known as *narrative listening* (Margulis et al., 2019). As the music unfolds, listeners project narrative structure onto its elements, relying on domain-general pattern recognition. Certain acoustic features tend to elicit specific narrative events, such as when high contrast and repetition evoke a chase (Margulis et al., 2022a).

For music to elicit imagined narratives also seems to require some form of common ground between composer and listener (cf. Clark, 1996). A composer’s musical choices are more legible to listeners who share the same culture-specific associations between sound and meaning, even centuries removed. For example, Americans report imagining more engaging narratives when listening to classical Western than Chinese music (Margulis et al., 2019), and their narratives share more similar content with each other than with those generated by Chinese listeners for the same pieces (Margulis et al., 2022b). Moreover, listeners struggle to match narratives from other cultures to the pieces that elicited them (McAuley et al., 2021).

For music generated artificially, listeners may experience even greater psychological distance from the so-called “composer.” People ascribe AI systems with less communicative intention than human artists (Mikalonyte & Kneer, 2022), and such impressions track decreased activity in cortical networks supporting mental-state attribution (Abu-Akel et al., 2020; Steinbeis & Koelsch, 2009). This may explain why AI-generated art is often evaluated less favorably than human works (Bellaiche et al., 2023; Shank et al., 2023), even when nearly indistinguishable from them (Chamberlain et al., 2018; Hong et al., 2022).

With AI-generated music infiltrating the modern soundscape, many listeners are aware that some of the music they encounter was artificially produced (NRG, 2024). AI composition, while sometimes disclosed explicitly, may otherwise be suspected (Rohrmeier, 2022). Even as AI output approaches or surpasses human compositional skill (Moura and Maw, 2021), believing that music was AI-generated may diminish its resonance with listeners. One potential consequence is reduced narrative listening: People may be less apt to imagine narratives when they believe a piece was AI generated as opposed to human composed, regardless of who—or what—*actually* composed the music.

We tested this possibility across two preregistered studies. In Study 1, participants listened to several pieces of instrumental music—all human composed, unbeknownst to them. For each piece, participants reported their narrative listening experience (Margulis et al., 2022b)—whether they imagined a story and how engaging it was—and rated the likelihood that the piece was composed by a human or a computer. We expected less narrative listening for pieces believed more likely to be computer composed. In Study 2, we experimentally manipulated participants’ beliefs by labeling human- and AI-composed pieces as either “Human” or “AI” composed. We expected that, above and beyond any influence of the actual composer, “AI”-labeled pieces would elicit less narrative listening than “Human”-labeled pieces.

## Study 1

### Method

#### Participants

We recruited 99 US adults from Amazon Mechanical Turk via CloudResearch (see Table 1). An additional 22 participants were excluded for failing attention checks or not completing all measures. The final sample provided > 97% power to detect small-to-medium effects (*d* = 0.4) in our main analyses.

**Table 1:** Participant Background Data

Variable	Study 1	Study 2
*N* (sampled / analyzed)	121 / 99	340 / 300
Gender (female / male)	41% / 59%	46% / 51%
Mean age in years (*SD*)	41 (12)	42 (12)
*Race/ethnicity*		
White	64%	71%
Black	15%	10%
Asian	8%	6%
Hispanic/Latinx	4%	4%
Multiracial	4%	4%
Other	5%	5%
*Mean years of experience (SD)*		
Playing an instrument	1.4 (2.4)	2.9 (5.4)
Music theory	0.7 (1.5)	1.3 (3.9)

### Materials

To assess narrative listening sans verbal cues, we selected six 60-s Western instrumental music clips, three classified as high narrativity and three as low narrativity by Margulis et al. (2019; see Table 2). Each piece’s MIDI file was sourced from MuseScore, a digital sheet music catalog with arrangements of classical music in the public domain. To minimize timbre differences and make the pieces seem plausibly AI generated, we used GarageBand software to render audio from each piece’s MIDI file. If the MIDI file encoded a certain set of orchestral instruments, we retained them in the final audio files exported from GarageBand. The six clips included two pieces with orchestral instrumentation (those by Beethoven and Schoenberg) and four with piano instrumentation (those by Ketelbey, Ravel, Mozart, and Debussy).

**Table 2:** Musical Stimuli (Margulis et al., 2019: live performance recordings; Study 1: MIDI versions)

Composer	Title	Narrativity (Margulis et al., 2019)	Narrativity (Study 1)
Ketelbey	*In the Mystic Land of Egypt*	High (.88)	.56
Ravel	*Gaspard de la Nuit*	High (.73)	.39
Beethoven	*Egmont Overture*	High (.68)	.57
Mozart	*Piano Sonata No. 7*. *II*	Low (.53)	.52
Schoenberg	*Suite, Op. 29*	Low (.43)	.34
Debussy	*En Blanc et Noir*	Low (.40)	.50

The Study 1 stimuli were MIDI versions of the human-composed pieces listed here. The Study 2 stimuli consisted of the Ketelbey, Ravel, Beethoven (modified from Study 1; see Study 2 Materials), and Mozart MIDI pieces, as well as an AI-generated counterpart for each piece. Narrativity scores are the proportion of “yes” responses to the Story Response Question (SRQ)

### Procedure

The study was administered via Qualtrics. First, participants read a passage that defined AI and described its ability to create impressive artwork. Next, they were instructed that they would listen to synthesized piano and orchestral music clips, each “composed by a computer or a human,” and answer questions about their listening experience. They were asked to listen naturally and not to specifically attempt to imagine a story (Margulis et al., 2019).

On the following screen, one randomly selected clip played automatically. After 60 s, participants could advance to the next screen. There they reported their familiarity with the piece (*yes/no*) and completed two established narrative listening measures: (a) a binary Story Response Question (SRQ) assessing *narrativity* (“Did you imagine a story when listening to the clip?”) and (b) four items assessing *narrative engagement* (NE; α = .94; e.g., “I imagined a vivid story”; 1, *strongly disagree*; 6, *strongly agree*), presented regardless of the SRQ response (Margulis et al., 2019). Next, participants answered two questions included for exploratory purposes. First, if they had reported imagining a story, they were asked to describe it in detail. If not, they were asked to speculate why. Second, participants rated the *communicative intention* behind the piece (“To what extent did you feel the music was trying to express something, such as an intention?”; 1, *not at all*; 6, *a great deal*; Steinbeis & Koelsch, 2009). Finally, participants expressed their belief about the composer’s identity (“Who do you think composed this piece?”; *computer*/*human*; order counterbalanced) and rated their confidence in this choice (0, *not at all*; 100, *extremely*).

The same series of questions was repeated for the remaining clips, presented in a randomized order. Following all clips, participants reported their general emotional reactions to music (Gold-MSI emotion subscale; Müllensiefen et al., 2014) and answered background questions.

### Results and discussion

Overall, the pieces were relatively unfamiliar to participants. The most recognized piece, by Ketelbey, was reported as familiar by just 14% of participants (across pieces: *M* = 10%, *SD* = 30%; see Supplementary Material for more detailed analyses of the familiarity data).

We computed participants’ confidence-weighted intuitions about composer identity by multiplying their choice on each trial (coded as -1, *computer*, or 1, *human*) by their confidence rating. Scores of -100 and 100 represent maximal confidence that the piece was computer composed and human composed, respectively.

**Fig. 1:**
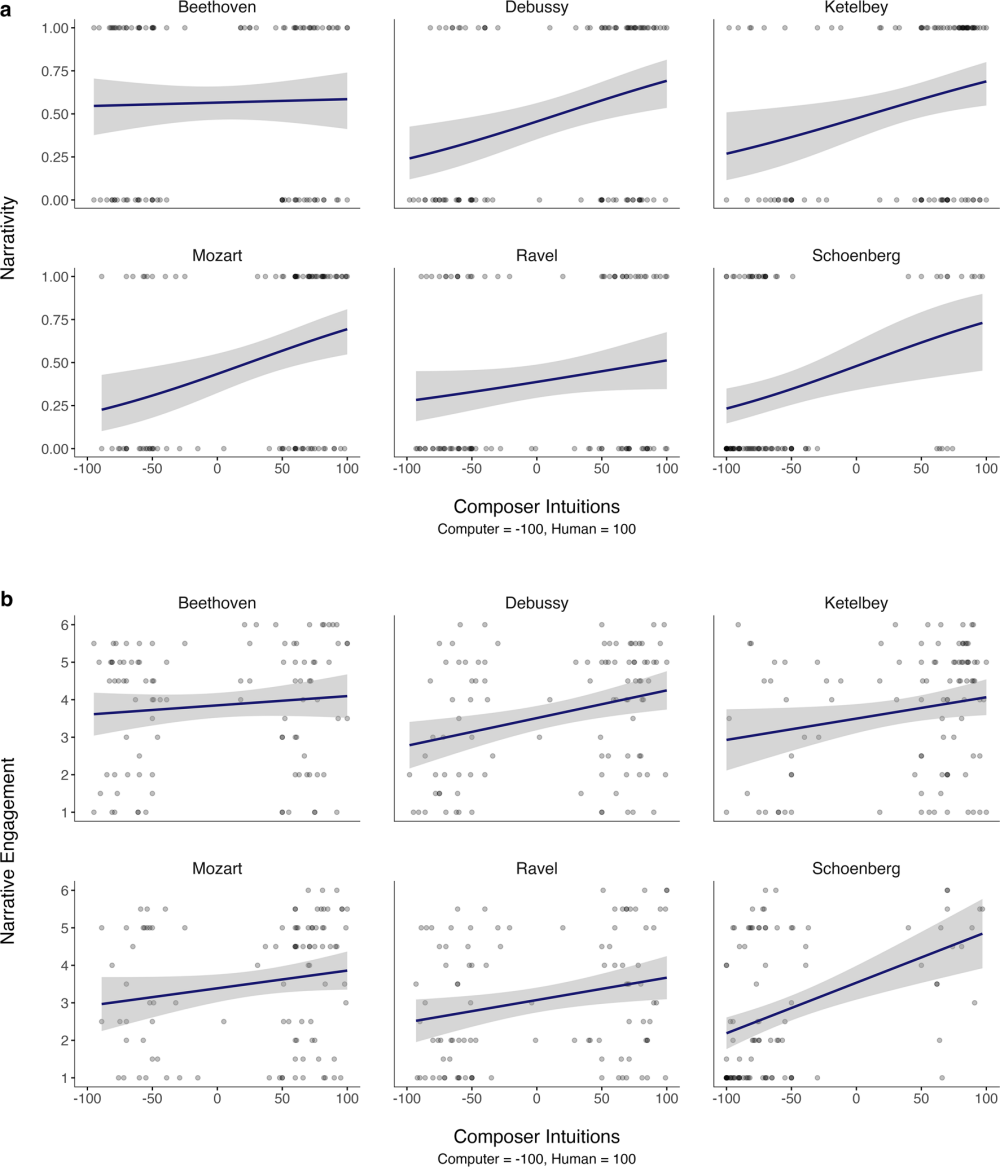
Relationship between composer intuition scores and **a** narrativity (SRQ) scores and **b** narrative engagement (NE) scores for each piece in Study 1. In **a**, each participant’s narrativity score was either 1 (imagined a story) or 0 (did not imagine a story). The shaded bands denote 95% CIs

Preregistered analyses assessed the relationship between these intuitions and narrative listening. As shown in Fig. 1a, composer intuition scores (*M* = 5.24, *SD* = 31.5) were positively associated with narrativity (*M* = 0.48, *SD* = 0.30; see Table 2 and Figure S1 in the Supplementary Material for narrativity and composer intuition data, respectively, by piece). The more strongly participants believed a piece was human composed, the more likely they were to imagine a story. Separate logistic regression analyses for each piece showed that composer intuitions accounted for significant variance in narrativity for four pieces (*p*s < .01), all but those by Beethoven and Ravel. For those pieces, the relationship was nominally in the same direction (*p*s > .1; see Supplementary Material for detailed analyses by piece). For the 76% of participants who reported imagining stories for some pieces but not others, the pieces that did not elicit stories (*M* = -16.50, *SD* = 69.50) were believed to be less likely human composed than the pieces that did (*M* = 25.40, *SD* = 65.40), *t*(74) = 6.00, *p* < .001, *d* = 0.65 (not preregistered).

As shown in Fig. 1b, composer intuition scores were also positively associated with narrative engagement (*M* = 3.43, *SD* = 1.10), computed by averaging the four NE ratings for each piece. For each participant, this relationship is captured by the unstandardized slope coefficient when regressing their six NE scores (one per piece) on their composer intuition scores. Across participants, the mean slope was positive (*M* = 0.007, *SD* = 0.01), *t*(98) = 4.62, *p* < .001, *d* = 0.47, indicating that the more strongly participants believed a piece was human composed, the more engaging their imagined stories were. In exploratory by-piece regression analyses, composer intuitions accounted for significant variance in narrative engagement for four pieces (*p*s < .05), all but those by Beethoven and Mozart (*p*s > .09; see Supplementary Material).

Additional exploratory analyses showed that participants’ communicative intention ratings predicted narrative listening when controlling for composer intuitions and participant characteristics, but composer intuitions no longer predicted narrative listening when controlling for communicative intention (see Supplementary Material). This suggests that the relationship between composer intuitions and narrative listening may be driven by ascribing less communicative intention to pieces believed to be AI composed.

The results of Study 1 are consistent with the possibility that believing music was AI composed suppresses narrative listening. However, the relationship could also run in the opposite direction: Imagining an engaging musical narrative may lead listeners to infer that the piece was more likely human composed. Alternatively, low-level acoustic properties could guide both composer intuitions and narrative listening. For example, atonal music may sound artificially produced and capture little narrative imagination (Steinbeis & Koelsch, 2009). These possibilities are not mutually exclusive but must be empirically disentangled.

Study 2 was designed to provide a direct test of the influence of composer beliefs. By manipulating the purported composer of a subset of pieces from Study 1, we assessed whether believing music was AI composed per se can suppress narrative listening. To instantiate such beliefs, we labeled each piece as “Human” or “AI” composed. Minimal labels of this sort have been shown to influence the perceived intentionality of music (Steinbeis & Koelsch, 2009), appreciation of visual art (Bellaiche et al., 2023; Chiarella et al., 2022), and absorption in literature (Messingschlager & Appel, 2024), among other attitudes and judgments (Flusberg et al., 2024). Moreover, the “AI” label may simulate the opaque experience of encountering real-world AI products, for which little else may be known (Bearman & Awajji, 2023).

A second, exploratory goal of Study 2 was to examine whether any effect of composer beliefs would be moderated by the *actual* composer of the music. The “AI” label might only suppress narrative listening, for example, when applied to AI-composed music, which often has distinctive properties such as anomalous pitches or excessive repetition (Rohrmeier, 2022). To investigate this possibility, Study 2 included both human-composed and AI-composed pieces, reflecting today’s increasingly AI-infused soundscape.

## Study 2

### Method

#### Participants

We recruited 300 participants, with an additional 40 excluded following the criteria of Study 1 (see Table 1 and Supplementary Material). This sample provided > 99% power to detect a small-to-medium effect of composer label (*η*^2^_p_ = .03) in our main analyses.

#### Materials

We expanded the number of stimuli to eight, four human composed and four AI composed. The human-composed clips were those by Beethoven, Ketelbey, Mozart, and Ravel, which a separate sample rated as plausibly AI composed (see Supplementary Material). To better match stimulus features across these clips, we re-exported the Beethoven MIDI file from Study 1 with piano instrumentation so that all four pieces would have piano timbre. We also increased the tempo of this clip to better match the corresponding clip in Margulis et al. (2019). To create AI-composed analogs, we used AIVA (2016) software, importing each human-composed clip as an “influence” and specifying the same tempo and key. The resulting clips had similar properties as their human-composed counterparts but were distinguishable as unique (see Supplementary Material).

#### Procedure

The procedure differed from Study 1 in three ways. First, the label “Composer: Human” or “Composer: AI” appeared centrally while each clip played. Second, on the following screen, participants completed only the SRQ, NE, and communicative intention measures, followed by questions assessing *general impressions* (i.e., five separate items assessing how enjoyable, pleasant, intense, moving, and surprising the piece was; 1, *not at all*; 6, *extremely*). These exploratory questions replaced the open-ended story descriptions, the Gold-MSI, and the familiarity question. The latter question was removed to mitigate demand characteristics from implying that some pieces might be familiar despite being “AI” labeled. Finally, the study used blocked randomization, with each of the four combinations of composer label and actual composer (i.e., “human”-labeled/human-composed clip, “AI”-labeled/human-composed clip, “human”-labeled/AI-composed clip, “AI”-labeled/AI-composed clip) presented once within the first four trials and once within the last four trials.

### Results and discussion

**Fig. 2:**
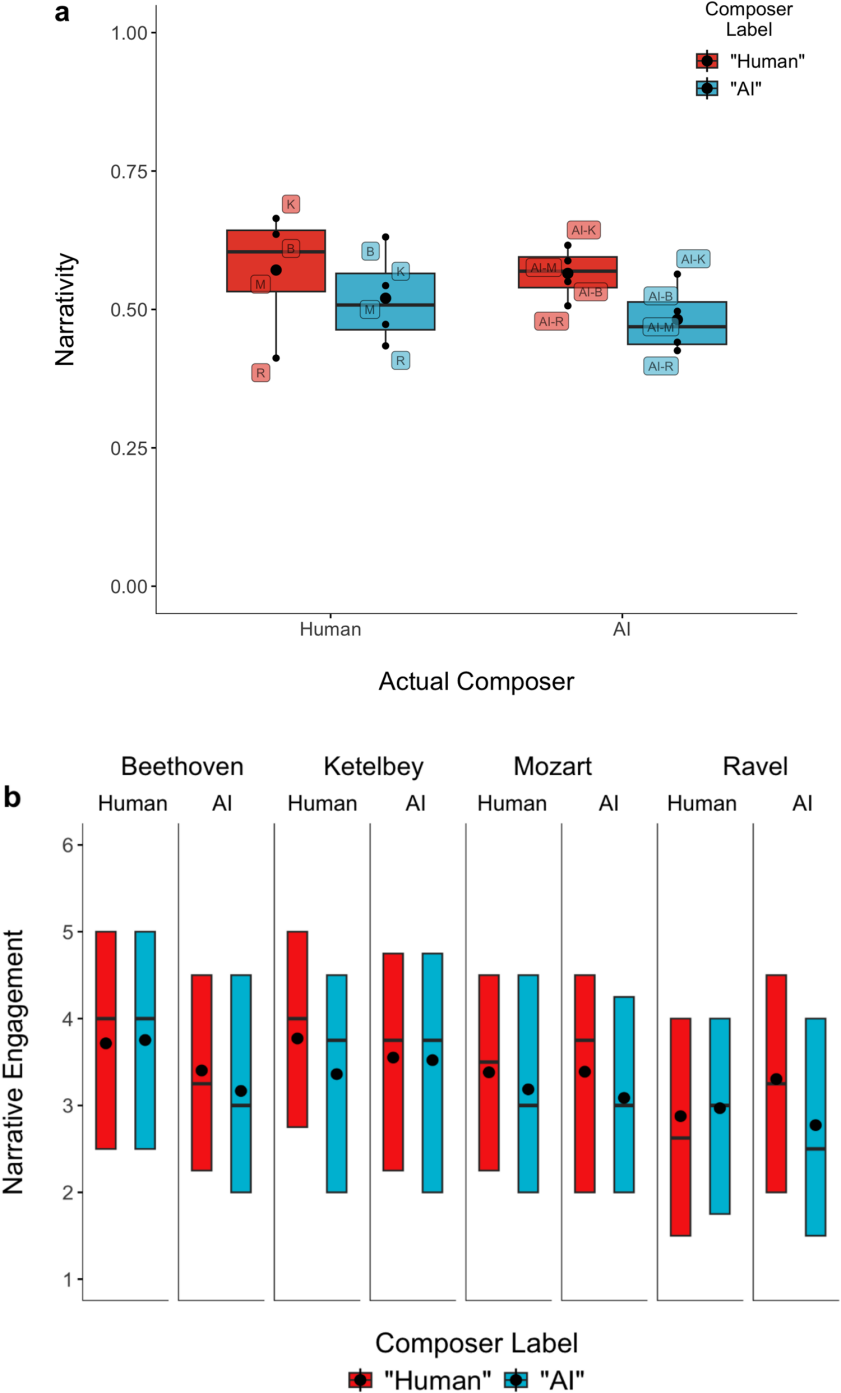
**a** Narrativity and **b** narrative engagement by composer label and actual composer for each piece in Study 2. Boxes denote the interquartile range (IQR), the middle line indicates the median, and black dots denote means. In **a**, pieces are denoted by the first letter of the composer’s last name (and by “AI-” for the AI-composed versions). In **b**, results are displayed for each human-composed piece alongside its AI-composed counterpart. The corresponding human composers are listed at the top

We used preregistered linear mixed-effects models (Baayen et al., 2008) with composer label (“Human”/“AI”), actual composer (human/AI), and their interaction as predictors of narrative listening. Each model began with a maximal random-effects structure, with slopes removed successively until the model converged (Barr et al., 2013). For narrativity, the final model included random intercepts for participants and pieces, as well as by-participant and by-piece random slopes for composer label. This model yielded a main effect of composer label, *F*(1, 6.36) = 12.67, *p* = .01, *η*^2^_p_ = .67, but no main effect of actual composer and no interaction (*p*s > .4). As shown in Fig. 2a, participants were less likely to imagine a story for AI-labeled pieces (*M* = .50, *SD* = .36) than human-labeled pieces (*M* = .57, *SD* = .37), regardless of the actual composer. For all clips except the actual Ravel composition, this difference aligned with the overall trend.

For narrative engagement, the final model included only random intercepts for participants and pieces. This model also yielded a main effect of composer label, *F*(1, 2091) = 21.03, *p* < .0001, *η*^2^_p_ = .01, with less narrative engagement for AI-labeled pieces (*M* = 3.23, *SD* = 1.15) than human-labeled pieces (*M* = 3.42, *SD* = 1.15; see Fig. 2b). For all clips except the actual Beethoven and Ravel compositions, this difference aligned with the overall trend. There was no main effect of actual composer, *F*(1, 6) = 0.25, *p* = .64, but the interaction approached significance, *F*(1, 2093) = 3.26, *p* = .07, *η*^2^_p_ = .002. As shown in Fig. 2b, the label effect was nominally larger for AI-composed than human-composed pieces. Exploratory analyses of participants’ general impressions of the music yielded results similar to those for narrative engagement (see Supplementary Material).

In other exploratory analyses, communicative intention was rated lower for AI-labeled than human-labeled pieces, and both communicative intention and general impressions strongly predicted narrative listening when controlling for composer label and other variables (see Supplementary Material). This suggests that inferences about communicative intention and impressions such as enjoyment and intensity may underlie the label effect we observed.

In two additional preregistered studies conducted prior to Study 2, we manipulated composer beliefs using extended cover stories (e.g., “An AI system generated this piece of music after being given many examples of Impressionist piano pieces [with an] exuberant, burbling sound”) rather than bare “human” and “AI” labels. These studies, detailed in the Supplementary Material, should be considered preliminary because they were underpowered, with a much smaller sample size or set of music stimuli than in Study 2. The effects of the cover stories on narrative listening were descriptively similar to those of the labels in Study 2 but mostly nonsignificant. Given the underpowered nature of these studies, these results should not be taken as evidence that bare labels are more effective than extended descriptions for instantiating beliefs about composer identity. That said, such descriptions risk being unconvincing if their details do not align with listeners’ impressions of the music—a possibility that could be evaluated in future work.

## General discussion

More and more of today’s music is the product of AI—and listeners are increasingly aware of it (NRG, 2024). We found that such awareness is closely tied to a core process of meaning-making from music: constructing a narrative while listening. In Study 1, participants reported imagining fewer and less engaging narratives while listening to music they regarded as more likely the output of a computer than a human mind. This relationship held even though such intuitions were often erroneous: All of the music was actually human composed. In Study 2, the same music elicited fewer and less engaging narratives when ascribed to AI than to a human composer, regardless of which label was correct. Together, these results suggest that attributing music to AI is associated with—and can engender—an impoverished listening experience, devoid of the mental narratives that unfold as the composer’s musical choices guide the listener’s imagination.

Our findings converge with evidence that music believed to be AI-generated elicits a variety of negative impressions (e.g., Agudo et al., 2022; Millet et al., 2023). Knowledge of AI generation need not be elaborate to shape the listening experience: Participants in Study 2 were not told exactly how the music was composed, only that the composer was human or AI. Our manipulation thus mirrored real-world encounters with AI-generated music, where the nature and extent of AI involvement may be unclear. In practice, a human composer might use AI as merely a *tool* for iterating through existing ideas or as a *creative partner* to suggest novel compositional choices (Louie et al., 2021; Suh et al., 2021). Future research should assess the impact of exposure to these increasingly common forms of human-AI creative collaboration (Mathur, 2025), emphasizing how AI’s role is framed (Chen et al., 2025; Kim et al., 2023) and gauging perceptions over time as AI becomes more integrated into society (Berker et al., 2006).

Several aspects of our findings also warrant further investigation. First, exploratory analyses suggested that the relationship between composer beliefs and narrative listening may have been driven by inferences about what the music was intended to communicate and by the extent to which the music was regarded as enjoyable, intense, and moving, among other impressions. Labeling music as AI composed, truthfully or otherwise, may lead listeners to infer that the music lacks meaning or intensity (Mikalonyte & Kneer, 2022) and therefore cannot support a narrative. This is consistent with evidence that the power of labeling often stems from reading between the lines of the label (Flusberg et al., 2024). Future work should examine which of many inferences triggered by the “AI” label—about lack of communicative intention, inspiration from lived experience (Messingschlager & Appel, 2025), or effort (Magni et al., 2023)—most strongly mediates its impact on narrative listening.

Second, some pieces yielded stronger effects than others, and the effect on narrative engagement in Study 2 was nominally stronger for actual AI compositions. These results suggest that participants were sensitive to acoustic markers of AI, which may help substantiate the “AI” label. Examining which acoustic features track composer beliefs and imagined narratives in real time—perhaps via behavioral indices of fluctuating perceptions (Margulis et al., 2022a) rather than retrospective self-report—would provide a fuller picture of how composer beliefs shape meaning-making. Behavioral measures might also circumvent the tendency to valorize distinctively human capacities (e.g., creativity) after learning about AI advances (Santoro & Monin, 2023). Additionally, although Study 2 controlled for expressive elements of the music such as phrasing and dynamics (Ansani et al., 2025), future research might manipulate such elements to examine the relative impact of composer beliefs and performance expressivity on narrative listening.

Finally, in an exploratory analysis detailed in the Supplementary Material, we found that the semantic content of participants’ imagined narratives did not depend on beliefs about composer identity. Semantic content may be too heavily constrained by culture-wide sound–meaning associations (Margulis et al., 2019) to be swayed by composer beliefs, even as such beliefs influence the frequency and intensity of the narratives. It is possible, however, that broader aspects of narrative content, such as coherence, would vary as a function of composer beliefs. Future research might also explore whether narratives elicited by purportedly AI-composed music evoke flatter or more negative emotional responses (cf. Margulis et al., 2022b)—consistent with the weaker general impressions elicited by AI-labeled music in Study 2, and with evidence that AI attribution inhibits art-induced awe (Millet et al., 2023).

As the virality of The Velvet Sundown illustrates, much AI-generated music may go undetected because it is designed to blur the distinctive qualities of human-composed works, yielding a kind of algorithmically curated easy listening (Bogost, 2025). With composer-rights organizations pushing for mandatory disclosure of AI usage (ASCAP, 2025), our findings suggest that truthful attribution can have real consequences for how music is perceived and understood. For music to inspire our inner storyteller, it helps to know there’s a human mind behind it.

## Supplementary Information

Additional file1 (DOCX 4251 KB)

## Data Availability

We have made all preregistrations, materials, and data available on the Open Science Framework (https://osf.io/8mdep/overview).
