# Supplementary material for: Is there a “mind” behind the music? Attributing music to AI can suppress narrative meaning-making
Source: Cogn Res Princ Implic. 2026 Mar 2;11:19. doi: 10.1186/s41235-026-00715-z (PMC12953833; doi:10.1186/s41235-026-00715-z)
Supplement: Supplementary file 1 — Additional file1 (DOCX 4251 KB) [file 41235_2026_715_MOESM1_ESM.docx]

**Supplementary Material**

**Study 1**

***Additional Methods***

Participants in both studies had ≥95% approval on ≥100 HITs on Amazon Mechanical Turk, and were compensated $2 for participating.

***Additional Results***

**Composer Intuitions.** Figure S1 shows composer intuition scores for each piece. The pieces by Ketelbey (*M* = 36.90, *SD* = 60.88) and Mozart (*M* = 29.22, *SD* = 62.17) were perceived as more likely to be human- than computer-composed (Ketelbey: *t*(98) = 6.03, *p* < .001; Mozart: *t*(98) = 4.68, *p* < .001), the piece by Schoenberg (*M* = -54.01, *SD* = 58.16) was perceived as more likely to be computer- than human-composed, *t*(98) = -9.24, *p* < .001, and the pieces by Debussy (*M* = 14.95, *SD* = 66.79), Ravel (*M* = 2.74, *SD* = 68.88), and Beethoven (*M* = 1.62, *SD* = 68.49) were perceived as about equally likely to be human- or computer-composed (*p*s > .05).


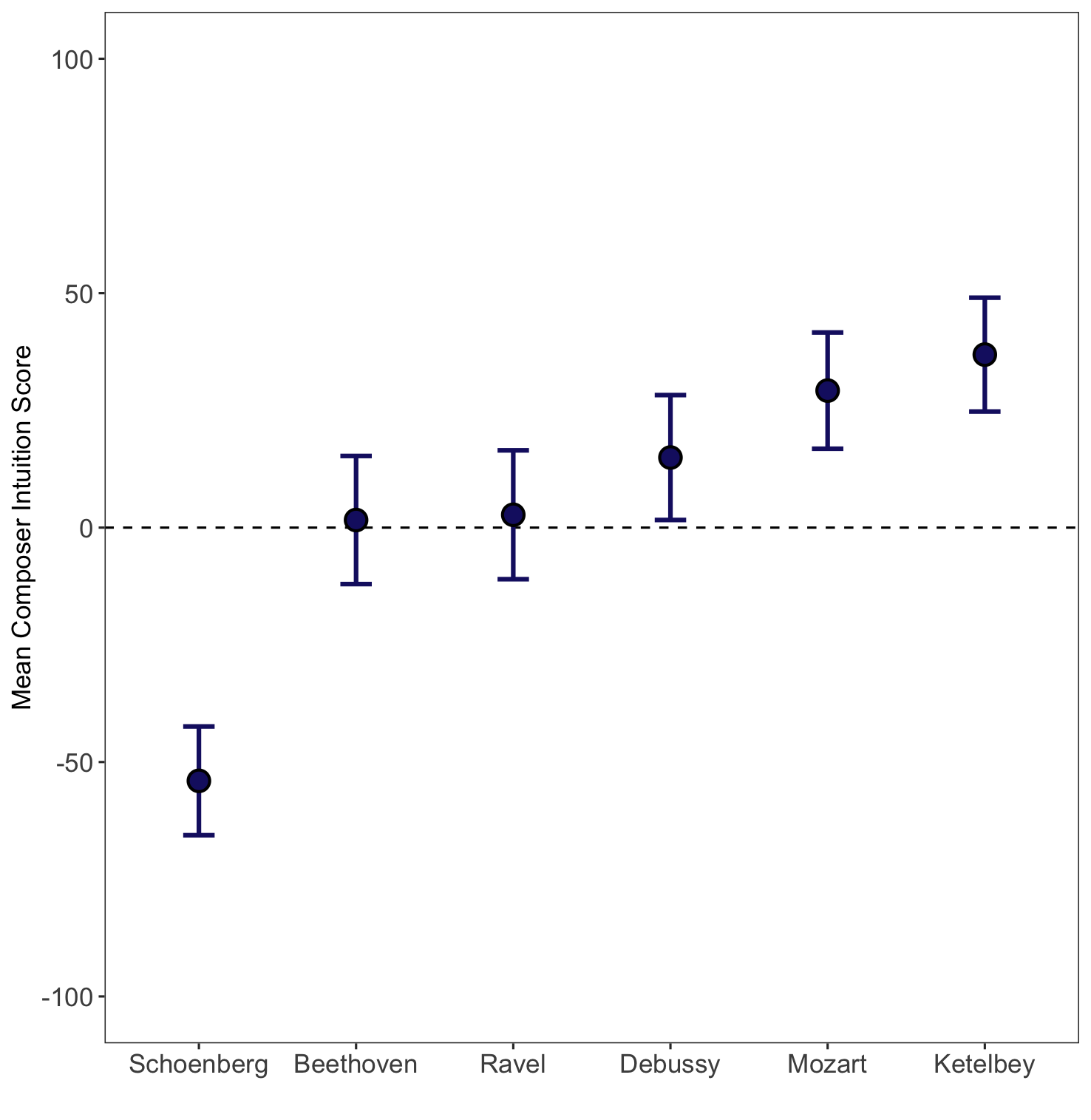


*Figure S1*. Composer intuition scores for each piece in Study 1. Scores of -100 and 100 represent maximal confidence that the piece was computer-composed and human-composed, respectively.

The dashed line indicates equal likelihood of human and computer attribution. Error bars represent 95% confidence intervals.

**By-Piece Regression Analyses.** As described in the main text, for each piece we conducted regression analyses predicting each measure of narrative listening (narrativity and narrative engagement) from composer intuition scores. Composer intuitions significantly predicted narrative listening for the pieces by Debussy (narrativity: *β*  = 1.28, *p* = .002; narrative engagement: *β*  = 0.30, *p* = .003), Ketelbey (narrativity: *β*  = 1.07, *p* = .01; narrative engagement: *β*  = 0.21, *p* = .04), and Schoenberg (narrativity: *β* = 1.37, *p* = .003; narrative engagement: *β*  = 0.40, *p* < .001). For the Beethoven piece, neither narrative listening measure was predicted by composer intuitions (narrativity: *β* = 0.11, *p* = .78; narrative engagement: *β* = 0.10, *p* = .31). For the Mozart piece, composer intuitions significantly predicted narrativity (*β*  = 1.30, *p* = .002), but not narrative engagement (*β*  = 0.17, *p* = .08). For the Ravel piece, composer intuitions significantly predicted narrative engagement (*β* = 0.24, *p* = .02), but not narrativity (*β* = 0.71, *p* = .09).

**Familiarity.** For each piece for which composer intuition scores significantly predicted narrativity, we conducted an exploratory logistic regression analysis predicting narrativity from both composer intuition scores and familiarity (whether or not the piece was familiar to the participant). Composer intuition scores remained a significant predictor for the pieces by Debussy (*β* = 1.27, *p* < .01), Ketelbey (*β* = 1.37, *p* < .01), and Mozart (*β* = 1.35, *p* < .01), but not for the piece by Schoenberg (*β* = 0.89, *p* = .09). In analogous linear regression analyses predicting narrative engagement, composer intuition scores remained a significant predictor for all four pieces: Debussy (*β* = 0.30, *p* = .003), Ketelbey (*β* = 0.21, *p* = .04), Ravel (*β* = 0.24, *p* = .02), and Schoenberg (*β* = 0.43, *p* < .001). These results suggest that the relationship between composer intuitions and narrative listening cannot be explained by participants’ familiarity with the pieces.

**Predictors of Narrative Listening.** Table S1 shows the results of exploratory regression analyses predicting each measure of narrative listening from composer intuition scores, communicative intention, familiarity, general emotional reactions to music (α = .79), music experience, and demographics.

**Table S1**

*Predictors of Narrative Listening in Study 1*

|  | **Narrativity** | | **Narrative Engagement** | |
| --- | --- | --- | --- | --- |
| **Predictor** | ***β*** | ***p*** | ***β*** | ***p*** |
| Composer intuitions | -0.03 | .73 | -0.09 | .32 |
| Communicative intention | **0.33** | **< .01** | **0.45** | **< .001** |
| Familiarity | 0.23 | .06 | **0.34** | **< .01** |
| Emotional reactions | 0.11 | .30 | 0.10 | .28 |
| Years playing instrument | -0.09 | .53 | -0.07 | .59 |
| Years of music theory | 0.01 | .94 | -0.05 | .71 |
| Age | -0.15 | .12 | **-0.24** | **< .01** |
| Gender | -0.14 | .16 | -0.09 | .27 |
| Race/ethnicity | 0.04 | .62 | 0.05 | .57 |

*Note*. Significant predictors are bolded. Gender was coded as 1 (female) or 0 (male). Due to the relatively small proportions of participants reporting various racial or ethnic identities (see Table 1 in the main text), race/ethnicity was coded as 1 (White) or 0 (non-White).

**Exploratory Semantic Content Analysis.** To assess the semantic content of participants’ imagined narratives, we used a sentence embedding model (all-MiniLM-L6-v2, built on SBERT; Reimers & Gurevych, 2019) to compute the cosine similarity score for each pair of narratives elicited by a given piece across participants. For each piece, we computed the mean cosine similarity score for (a) pairs for which both pieces were believed to be human-composed (based on the binary composer identity question), (b) pairs for which both pieces were believed to be AI-generated, and (c) pairs for which one piece was believed to be human-composed and the other was believed to be AI-generated. As shown in Table S2, narratives were no more similar in content when generated by participants who shared the same intuitions about the composer of the piece (both human or both AI) than by those who held different intuitions (one human, one AI); repeated-measures ANOVA: *F*(2, 5) = 0.50, *p* = .62.

**Table S2**

*Mean Cosine Similarity Scores (with Standard Deviations) by Piece in Study 1*

| **Composer** | **Human-Human** | **AI-AI** | **Human-AI** |
| --- | --- | --- | --- |
| Beethoven | 0.25 (0.12) | 0.23 (0.13) | 0.25 (0.13) |
| Debussy | 0.26 (0.14) | 0.25 (0.15) | 0.24 (0.14) |
| Ketelbey | 0.22 (0.13) | 0.20 (0.12) | 0.20 (0.13) |
| Mozart | 0.24 (0.15) | 0.20 (0.13) | 0.22 (0.15) |
| Ravel | 0.26 (0.12) | 0.24 (0.13) | 0.25 (0.14) |
| Schoenberg | 0.21 (0.10) | 0.26 (0.12) | 0.24 (0.12) |
| **All Pieces** | **0.23 (0.13)** | **0.21 (0.12)** | **0.22 (0.13)** |

**Study 2**

***Additional Methods***

**Participants.** In Study 2, the background questionnaire included questions about knowledge and experience with AI. Participants reported a moderate extent of knowledge about generative AI before the study (*M* = 3.8, *SD* = 1.2; 1 = *nothing*, 6 = *a great deal*). The majority of participants (65%) had used AI tools in the past. Of those with experience using AI tools, 11% reported daily use, 28% reported weekly use, and 22% reported monthly use; 16% reported using AI tools less often than monthly, and 23% reported using them just once or a few times ever.

**Materials.** To determine the four pieces for Study 2, we recruited a separate group of participants from Amazon Mechanical Turk (*N* = 50). After listening to each of the six clips from Study 1, participants rated how believable it was that the music was generated by an “AI music composer” (1 = *not at all believable*; 6 = *completely believable*), among other attributes of the music. All of the clips were rated as believably AI-composed, with mean ratings between 4 and 5. We selected the clips by Beethoven (*M* = 4.32, *SD* = 1.38), Ketelbey (*M* = 4.36, *SD* = 1.31), Mozart (*M* = 4.60, *SD* = 1.26), and Ravel (*M* = 4.40, *SD* = 1.36), dropping the Debussy clip (*M* = 4.32, *SD* = 1.36) to avoid including a second Impressionist piece (besides Ravel) and the Schoenberg clip (*M* = 4.90, *SD* = 1.16) because it was rated as considerably more AI-sounding than the others. After stimulus selection, we modified the Beethoven clip so that it would have the same instrumentation (solo piano) as the other pieces. Table S3 shows features of the four final human-composed clips and their AI-composed analogs used in Study 2.

**Table S3**

*Features and Mean Ratings (with Standard Deviations) of Study 2 Stimuli*

| **Composer** | | **Key** | **Tempo** | **Communicative Intention** | **General Impressions** |
| --- | --- | --- | --- | --- | --- |
| Beethoven | Original | F Minor | 160 bpm | 4.51 (1.23) | 4.17 (0.42) |
|  | AI |  | 140 bpm | 3.97 (1.34) | 3.66 (0.26) |
| Ketelbey | Original | D Minor | 100 bpm | 4.26 (1.25) | 3.87 (0.33) |
|  | AI |  | 95 bpm | 4.14 (1.36) | 3.84 (0.27) |
| Mozart | Original | F Major | 115 bpm | 3.85 (1.24) | 3.51 (0.48) |
|  | AI |  |  | 3.87 (1.26) | 3.46 (0.37) |
| Ravel | Original | E-flat Minor, with a B-flat ostinato | 105 bpm | 3.51 (1.44) | 2.99 (0.19) |
|  | AI | Bb Minor | 115 bpm | 3.57 (1.35) | 3.18 (0.24) |

*Note.* For each composer, “Original” refers to the composition by that composer and “AI” refers to its AI-generated counterpart. All pieces had solo piano instrumentation. In Study 1, the pieces by Beethoven and Schoenberg had orchestral instrumentation; the latter piece was not included in Study 2.

***Additional Results***

**General Impressions.** To analyze participants’ general impressions of the music (how enjoyable, pleasant, intense, moving, and surprising it was; α = .87), we used an exploratory mixed-effects model analogous to those for the narrative listening measures (see main text). The final model assessing composite general impression ratings included random intercepts for participants and pieces. This model yielded a main effect of composer label, *F*(1, 2091) = 21.69, *p* < .0001, *η*^2^_p_ = .01, no main effect of actual composer, *F*(1, 6) = 0.12, *p* = .74, and no significant interaction, *F*(1, 2091) = 3.22, *p* = .07, *η*^2^_p_ = .002. Similar to the results for narrative engagement, participants reported weaker reactions to the pieces (i.e., less enjoyment, intensity, etc.) when they were AI-labeled (*M* = 3.50, *SD* = 1.19) than human-labeled (*M* = 3.66, *SD* = 1.14), and this difference was nominally larger for AI-composed than human-composed pieces (see Figure S2).


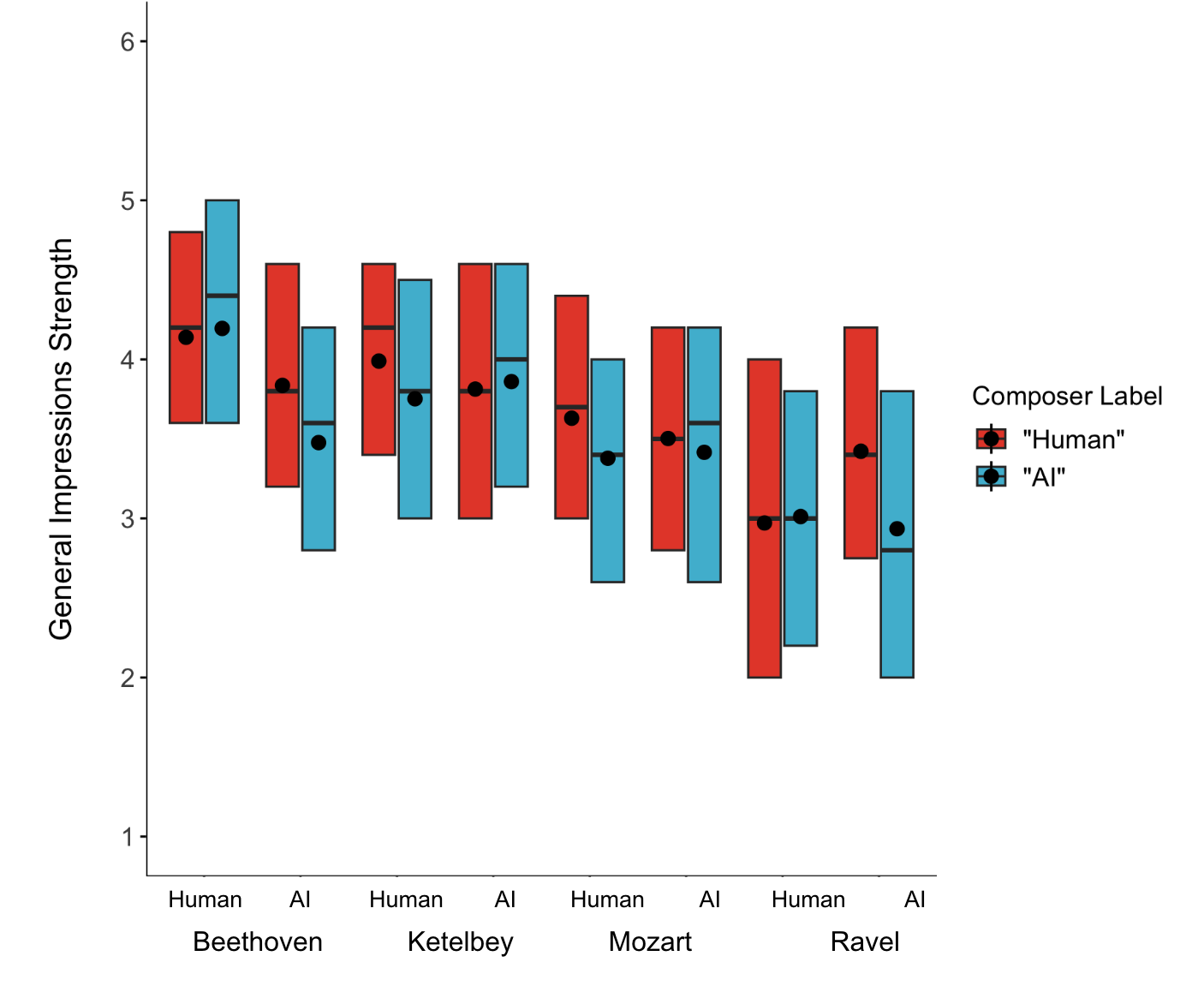


*Figure S2.* Strength of general impressions by composer label and actual composer (human or AI; x-axis) for each piece in Study 2. Boxes denote the interquartile range (IQR), the middle line indicates the median, and black dots denote means.

**Communicative Intention.** To analyze participants’ communicative intention ratings, we used an exploratory mixed-effects model analogous to those for the narrative listening and general impression measures. The final model included random intercepts for participants and pieces. This model yielded a main effect of composer label, *F*(1, 2091) = 65.77, *p* < .0001, *η*^2^_p_ = .03, with communicative intention rated higher for human-labeled pieces (*M* = 4.14, *SD* = 1.25) than AI-labeled pieces (*M* = 3.78, *SD* = 1.41). There was no main effect of actual composer, *F*(1, 6) = 0.33, *p* = .59, and no interaction, *F*(1, 2091) = 1.96, *p* = .16.

**Predictors of Narrative Listening.** Table S4 shows the results of exploratory linear mixed-effects models predicting each measure of narrative listening from composer label, actual composer, communicative intention, general impressions, music experience, and demographics.

**Table S4**

*Predictors of Narrative Listening in Study 2*

|  | **Narrativity** | | **Narrative Engagement** | |
| --- | --- | --- | --- | --- |
| **Predictor** | ***β*** | ***p*** | ***β*** | ***p*** |
| Composer label | 0.01 | .30 | 0.01 | .69 |
| Actual composer | -0.01 | .81 | 0.01 | .77 |
| Communicative intention | **0.12** | **< .001** | **0.39** | **< .001** |
| General impressions | **0.15** | **< .001** | **0.60** | **< .001** |
| Years playing instrument | 0.02 | .34 | -0.04 | .53 |
| Years of music theory | -0.04 | .08 | -0.03 | .65 |
| Age | -0.02 | .21 | -0.09 | .08 |
| Gender | -0.01 | .69 | 0.02 | .81 |
| Race | -0.02 | .59 | -0.07 | .50 |

*Note*. Significant predictors are bolded. Gender was coded as 1 (female) or 0 (male). Due to the relatively small proportions of participants reporting various racial or ethnic identities (see Table 1 in the main text), race/ethnicity was coded as 1 (White) or 0 (non-White).

**Study S1**

We conducted two additional studies (denoted as Studies S1 and S2) in which we experimentally manipulated composer beliefs using cover stories rather than labels. These studies were carried out between the two studies reported in the main text, and their methods had elements of each one. As discussed in the main text and detailed below, however, these studies were underpowered for detecting small-to-medium effects of purported composer. For each of the additional studies, we report by-participant and by-piece analyses of narrative listening responses.

***Method***

**Participants.** We recruited 97 participants following the criteria of Study 1. An additional 24 participants were excluded for failing attention checks or not completing all measures. The final sample provided only 50% power to detect a small-to-medium effect of purported composer (*d* = 0.4) in our by-participant analyses.

**Materials and Procedure.** The materials were the same six clips used in Study 1. The procedure was identical to Study 2, with three exceptions. First, each clip was preceded by a cover story rather than accompanied by a composer label. The cover story was presented on the screen before the clip and framed the piece as human- or AI-composed (e.g., “[A human composer wrote/An AI system generated] this piece of music after [hearing/being given] many examples of Impressionist piano pieces. [He/It] identified patterns among the different pieces to emulate the same exuberant, burbling sound.”). For each participant, three randomly-selected clips were framed as human-composed and the other three were framed as AI-composed. Clips of each type were randomly intermixed. Second, participants did not rate their general impressions of the pieces. Third, at the end of the study, participants reported how much they believed the cover stories (“On the whole, to what extent did you believe the descriptions that preceded each piece of music you listened to?”; 1 = *not at all*; 6 = *completely*) in addition to completing the demographic and background questions.

***Results***

**Narrativity.** Narrativity was nominally greater for pieces framed as human-composed (*M* = .56, *SD* = .35) than for pieces framed as AI-composed (*M* = .51, *SD* = .35). This difference approached significance in the by-participant analysis, *t*(96) = 1.85, *p* = .07, *d* = 0.19, but not in the by-piece analysis, *t*(5) = 1.06, *p* = .34.

**Narrative Engagement.** Similar to narrativity, narrative engagement was nominally greater for purportedly human-composed pieces (*M* = 3.58, *SD* = 1.23) than for purportedly AI-composed pieces (*M* = 3.39, *SD* = 1.30). This difference approached significance in the by-participant analysis, *t*(96) = 1.91, *p* = .06, *d* = 0.19, but not in the by-piece analysis, *t*(5) = 0.93, *p* = .40.

**Study S2**

***Method***

**Participants.** We recruited 298 participants following the criteria of Study 1. An additional 42 participants were excluded for failing an attention check or not completing all measures. The final sample provided 93% power to detect a small-to-medium effect of purported composer (*d* = 0.4) in our by-participant analyses, assuming that participants’ mean scores on the narrative listening measures were reliable. This assumption may be unwarranted, however, because participants only listened to two music clips per purported composer, half as many as in Study 2. Given that overall narrativity and narrative engagement varied considerably across pieces in Studies 1 and 2 (see Table 2, Figure 1, and Figure 2 in the main text), the small number of clips likely yielded substantial noise in mean scores, reducing the power of the study relative to Study 2.

**Materials and Procedure.** The methods were identical to Study S1, with three exceptions. First, each participant listened to the four clips of human-composed music used in Study 2, with two framed as human-composed and two framed as AI-composed. Second, each clip was preceded by a cover story similar to those used in Study S1, but with slightly more information about the AI composition process. For example, the human-framed piece by Beethoven was preceded by “The composer of the following piece envisioned certain melodies and chords that sound dramatic and intense, and then he used software to notate them,” whereas the AI-framed counterpart was preceded by “In response to a user’s request for ‘dramatic, intense music,’ an AI system used machine learning algorithms to analyze patterns from human-composed music to generate the following piece.” Third, participants reported their general impressions of the music as in Study 2.

***Results***

**Narrativity.** Narrativity did not differ significantly between pieces framed as human-composed (*M* = .61, *SD* = .49) and pieces framed as AI-composed (*M* = .59, *SD* = .49) in either the by-participant analysis, *t*(274) = 0.72, *p* = .50, or the by-piece analysis, *t*(3) = .56, *p* = .60 (not preregistered). A preregistered chi-square test of independence yielded the same outcome: the number of pieces for which participants imagined a story did not differ by purported composer (human or AI), ꭓ^2^(2) = 0.03, *p* > .99.

**Narrative Engagement.** Narrative engagement was significantly greater for purportedly human-composed pieces (*M* = 3.61, *SD* = 1.61) than for purportedly AI-composed pieces (*M* = 3.48, *SD* = 1.61) in the by-participant analysis, *t*(273) = 2.30, *p* = .02, *d* = 0.14, but not in the by-piece analysis, *t*(3) = 1.40, *p* = .30 (not preregistered), despite a medium-to-large effect size (*d* = 0.70). We also conducted a preregistered linear mixed-effects analysis predicting narrative engagement from purported composer, with random intercepts for participants and pieces. This model yielded a significant effect of purported composer as well, *F*(1, 833.9) = 4.54, *p* = .03, *η*^2^_p_ = .005.

# References

Reimers, N., & Gurevych, I. (2019). Sentence-BERT: Sentence embeddings using Siamese BERT-Networks. In K. Inui, J. Jiang, V. Ng, & X. Wan (Eds.), *Proceedings of the 2019 Conference on Empirical Methods in Natural Language Processing and the 9th International Joint Conference on Natural Language Processing (EMNLP-IJCNLP)* (pp. 3982–3992). Association for Computational Linguistics.https://doi.org/10.18653/v1/D19-1410
